# Fusion-Assisted Hydrothermal Synthesis of Technogenic-Waste-Derived Zeolites and Nanocomposites: Synthesis, Characterization, and Mercury (II) Adsorption

**DOI:** 10.3390/ijms241411317

**Published:** 2023-07-11

**Authors:** Madina Suleimenova, Saule Zharylkan, Meruyert Mekenova, Alibek Mutushev, Seytkhan Azat, Aidana Tolepova, Alzhan Baimenov, Aliya Satayeva, Zhandos Tauanov

**Affiliations:** 1 Faculty of Chemistry and Chemical Technology, Al-Farabi Kazakh National University, Almaty 050040, Kazakhstan; 2 LLP Scientific Production Technical Center “Zhalyn”, Almaty 050012, Kazakhstan; 3 Laboratory of Engineering Profile, Satbayev University, Almaty 050013, Kazakhstan; 4 National Laboratory Astana, Nazarbayev University, Astana 010000, Kazakhstan

**Keywords:** zeolites, nanocomposites, fusion synthesis, silver, magnetite, mercury removal

## Abstract

This study presents the synthesis of zeolites derived from coal fly ash (CFA) using the fusion-assisted alkaline hydrothermal method. The zeolites were synthesized by combining CFA and NaOH at a molar ratio of 1:1.2 under fusion temperatures of 500, 600, and 700 °C. Subsequently, the obtained zeolites were subjected to further modifications through the incorporation of magnetic (Fe_3_O_4_) and silver (Ag^0^) nanoparticles (NPs). The Fe_3_O_4_ NPs were introduced through co-precipitation of Fe(NO_3_)_2_ and FeCl_3_ at a molar ratio of 1:1, followed by thermal curing at 120 °C. On the other hand, the Ag^0^ NPs were incorporated via ion exchange of Na^+^ with Ag^+^ and subsequent reduction using NaBH_4_. The synthesized porous materials exhibited the formation of zeolites, specifically analcime and sodalite, as confirmed by X-ray diffraction (XRD) analysis. Additionally, the presence of Fe_3_O_4_ and Ag^0^ NPs was also confirmed by XRD analysis. The elemental composition analysis of the synthesized nanocomposites further validated the successful formation of Fe_3_O_4_ and Ag^0^ NPs. Nitrogen porosimetric analysis revealed the formation of a microporous structure, with the BET surface area of the zeolites and nanocomposites ranging from 48.6 to 128.7 m^2^/g and pore sizes ranging from 0.6 to 4.8 nm. The porosimetric characteristics of the zeolites exhibited noticeable changes after the modification process, which can be attributed to the impregnation of Fe_3_O_4_ and Ag^0^ NPs. The findings of this research demonstrate the effectiveness of the fusion-assisted method in producing synthetic zeolites and nanocomposites derived from CFA. The resulting composites were evaluated for their potential application in the removal of mercury ions from aqueous solutions. Among the samples tested, the composite containing Ag^0^ NPs exhibited the highest adsorption capacity, reaching 107.4 mg of Hg^2+^ per gram of composite. The composites modified with Fe_3_O_4_ NPs and Ag/Fe_3_O_4_ nanocomposites displayed adsorption capacities of 68.4 mg/g and 71.4 mg/g, respectively.

## 1. Introduction

Contamination of water bodies with heavy metal ions is one of the alarming issues that the world faces in this century. The major fraction of fresh and surface water contamination by heavy metals is caused by coal-fired power plants followed by agricultural, municipal, and industrial waste products [1,2,3]. According to Kumar and colleagues [4], the concentration of some heavy metals exceeds the average permissible limits recommended by the WHO and US EPA, which creates adverse effects on the environment. Among these metals, mercury and its species are considered the most toxic and dangerous metals as they cause harmful effects on the neurological system and fertility of adults, as well as adversely affecting infants, even at minimal concentrations [5,6]. These global concerns triggered a more detailed study of this metal and its species, which eventually resulted in the signing of the Minamata Convention between 127 countries and one regional economic integration organization [7,8,9,10]. According to this convention, the signed parties must adhere to the minimum concentration range of mercury in the environment (water, soil, air), meticulously control both the prevention of its release, and apply the most efficient ways of remediation. There are several technologies commonly applied to the remediation of mercury from water, which include adsorption, membrane separation, ion exchange, and bioremediation [5,11]. 

Another important issue that is faced by many countries is the proper utilization of coal fly ash (CFA) accumulated from thermal power plants. One possible use of this low-cost raw material is an application in the synthesis of porous zeolites since they have similar chemical compositions. This allows the conversion of amorphously structured CFA into crystalline-structured zeolites that are generally produced via an alkaline hydrothermal treatment [12,13,14]. More recently a combination of the hydrothermal method with fusion, ultrasound, or microwave treatments has become prevalent. This allows for the enhancement of the physical and chemical properties of CFA-derived zeolites, and minimizes the synthesis time and costs. In addition, zeolites can be further enhanced by applying external or internal structure modifications. Table 1 presents results of selected research into applying fusion or a combination of fusion with other synthesis methods for the production of CFA-derived zeolites for the removal of heavy metals from water. 

As shown, there are many studies on the removal of heavy metals from water; however, limited studies are available on mercury remediation using CFA-derived zeolites. In addition, most of the studies are restricted to CFA-derived zeolites without further external or internal modifications to enhance the targeted removal performance, and there are fewer investigations on the mechanisms of the removal of mercury ions from water. Tauanov et al. [22,23] tried to fill this gap and meticulously examined the CFA-derived zeolites and composites impregnated with silver nanoparticles (Ag^0^ NPs). The authors used a hydrothermal synthesis method and examined the mercury speciation under adsorption conditions using speciation software, assessed the adsorption kinetics, and constructed isotherm models. However, although it is another interesting research topic, to the best of our knowledge, there are few related studies in the literature on fusion-assisted hydrothermal synthesis of zeolites that are further internally modified with nanoparticles, and have been examined for removal of mercury from water. According to our knowledge, the impregnation of a small quantity of Ag^0^ NPs may substantially increase the removal efficiency of Hg^2+^ from water with comparably improved stability under strong acidic and neutral conditions suitable for industrial applications [24]. Moreover, the adsorbents may be modified with magnetic nanoparticles to improve the separation after purification. 

In this work, a local CFA sample was used for the synthesis of zeolites via fusion-assisted alkaline hydrothermal treatment, which allows minimization of the synthesis time and enhancement of the porosimetric properties as compared to conventional hydrothermal method. Synthetic zeolites were modified by impregnation of silver to increase the affinity towards mercury and magnetite nanoparticles (Fe_3_O_4_ NPs) to improve the separation via ion exchange and co-precipitation methods, followed by reduction within a porous structure. The materials were characterized by advanced characterization methods for identification of their zeolitic phase, surface morphology, chemical composition, and porosimetric characteristics. Synthetic zeolites and nanocomposites were examined for the removal of mercury (II) ions from water via batch adsorption kinetics. A possible adsorption mechanism was proposed based on adsorption performance, characterization results, and previously generated speciation studies. 

## 2. Results and Discussion

### 2.1. Characterization

The chemical composition of the parent E-CFA and E-ZFAs synthesized at different fusion temperatures is presented in Table 2. As shown, the major fraction of E-CFA is aluminosilicates (a total of 61.119 wt.%), with a Si/Al ratio of about 3.31. These chemical characteristics, in combination with relatively low amounts of Ca (6.499 wt.%), Mg (0.140 wt.%), and S (0.274 wt.%), indicate that the coal origin is bituminous; this corresponds to CFA Type F, which is typical of local CFA samples [25,26]. The Fe content (22.275 wt.%), on the other hand, is substantially higher than in other regions of the world, which generally varies in the range of 2.6–19.3 wt.% [22,27]. Since one of the aims of this study is to provide a magnetic property to the zeolitic composite via impregnation of magnetite for easy separation, an inherent presence of iron sources is considered advantageous. The chemical composition of synthetic zeolites significantly changed upon alkaline fusion followed by hydrothermal treatment. The zeolitic microstructure is enriched with sodium (6.326–9.978 wt.%), which is readily available for ion exchange reactions with mercury ions in water. The total fraction of aluminosilicates varies between 45.495 and 47.124 wt.%, which acts as the zeolitic nucleation framework and growth source during the conversion of amorphous E-CFA into crystalline structured zeolites. A slight increase in the aluminosilicate fraction of E-ZFA-700 and lower amounts of Na and O might be the sign of phase transition, wherein the E-CFA is converted to other types of synthetic zeolites due to elevated temperature. 

**Figure 1:**
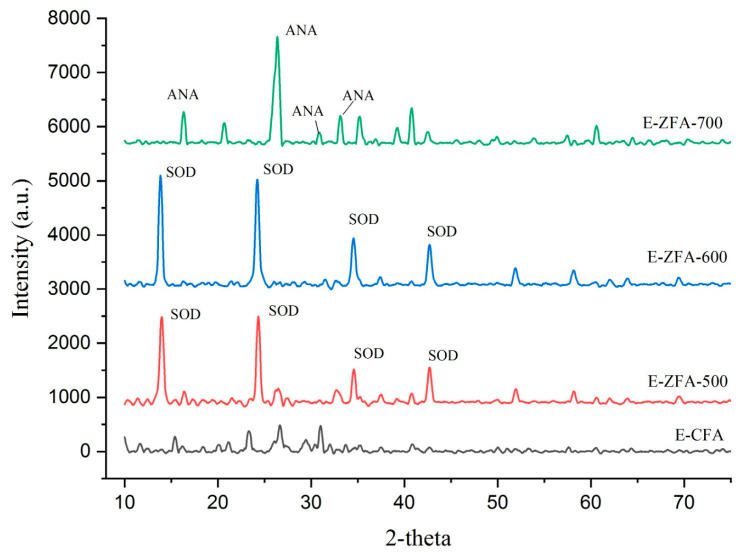
XRD spectra of the parent E-CFAs and E-ZFAs produced at different fusion temperatures.

The mineralogical phase composition of the parent E-CFAs and E-ZFAs produced under various alkaline fusion conditions was studied using an X-ray diffractometer to identify the formed zeolitic framework structures as shown in Figure 1. According to results, the main phases present in the amorphous structure of the E-CFA are mullite, quartz, and hematite, which is in corroboration with our previous studies and literature data [28,29]. Upon elevated alkaline fusion treatment followed by hydrothermal reaction, the amorphous phase of the parent E-CFA is converted to a crystalline zeolitic framework of sodalite (SOD) and analcime (ANA). The results of XRD patterns corroborate with the JCPDS Card: 76-090 and RRUFF ID: R060023.1 for analcime, and JCPDS Card: 75-0709 and RRUFF ID: R040141.1 for sodalite. 

It is interesting to note that zeolites synthesized under fusion temperatures of 500 °C and 600 °C developed a zeolitic phase that is comparably pure from the initial components (mullite, quartz) and trace amounts of other zeolites. A relatively higher intensity of the E-ZFA-600 suggests that a more crystalline and porous sodalite framework is produced. Zeolites produced under a fusion treatment of 700 °C were able to produce E-ZFA-700, which developed an analcime zeolitic framework. This mineralogical information linked with a chemical composition analysis evidences the phase transition of the E-CFA due to the evaporation of sodium (Na) at higher melting temperatures towards a more stable phase [30,31]. However, in the attempt to produce a more stable zeolitic phase, one inevitably faces the admixtures of other zeolitic phases, which in turn might be not suitable for certain applications. In light of this knowledge, and to attempt to control the zeolitic framework variations, this research continued modifications with the SOD framework E-CFA-600 zeolite that was synthesized under a fusion temperature of 600 °C with relatively high purity.

**Figure 2:**
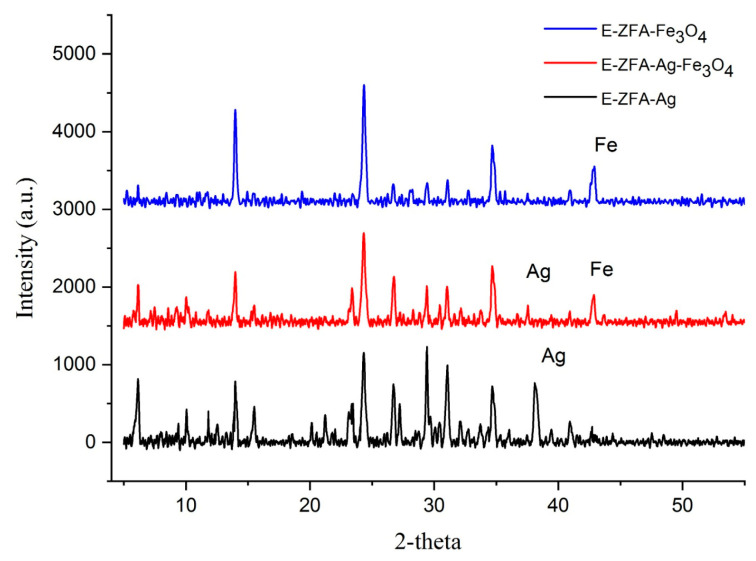
XRD spectra of the modified nanocomposites.

The XRD patterns of silver-, magnetite-, and silver-magnetite-modified nanocomposites are presented in Figure 2. The sample E-ZFA-Ag contains several peaks of a crystalline structure of zeolite, with an additional peak at 38.29° assigned to the (111) plane of Ag^0^ NPs. Both E-ZFA-Fe_3_O_4_ and E-ZFA-Ag-Fe_3_O_4_ have pronounced peaks of Fe_3_O_4_ NPs (311). Further data on mineralogy, morphology, and chemical composition of parent CFAs, synthesized zeolites, and nanocomposites can be found elsewhere [22,25].

The elemental composition of E-ZFA-Ag, E-ZFA-Fe_3_O_4_, and E-ZFA-Ag-Fe_3_O_4_ composites is presented in Table 3. According to the results, all elements of the zeolitic scaffold are on the same level for all composites. The main differences are in the content of silver and iron elements. In the case of E-ZFA-Ag, the Fe content in the structure is around 6%, while the modification with silver provided the opportunity to attach ~2.3% of Ag. The E-ZFA-Fe_3_O_4_ consists of 11.5% of iron due to modification of the zeolite surface with Fe_3_O_4_. The use of a mixture of Ag and Fe increased the percentage of these elements to 1.2 and 8.6%, respectively.

**Figure 3:**
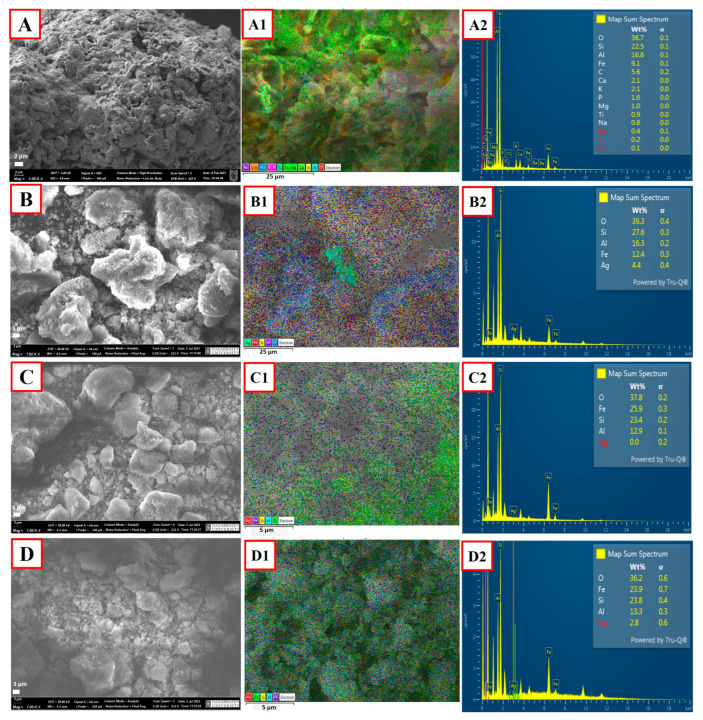
SEM micro-images and EDS mapping of E-ZFA (**A**–**A2**), E-ZFA-Ag (**B**–**B2**), E-ZFA-Fe_3_O_4_ (**C**–**C2**), and E-ZFA-Ag-Fe_3_O_4_ (**D**–**D2**).

**Figure 4:**
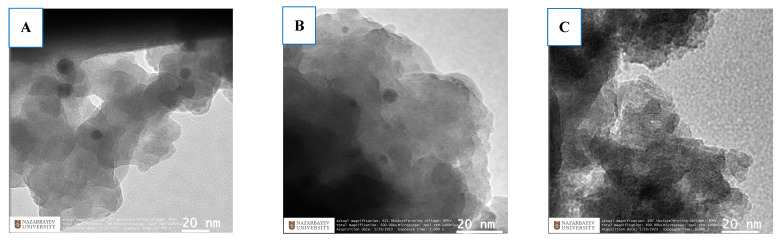
TEM analysis of the E-ZFA-Ag (**A**), E-ZFA-Ag-Fe_3_O_4_ (**B**), and E-ZFA-Fe_3_O_4_ (**C**).

Surface morphology and elemental analysis of the parent E-ZFA-600 and modified E-ZFAs using scanning electron microscopy demonstrate a porous structure of all zeolites (Figure 3A–D). The elemental composition of bare zeolite consists of various elements, including Fe of an average 9.1 wt.% with no silver inside (Figure 3(A1,A2)). Modification of E-ZFA with Ag^0^ NPs was able to increase the percentage of silver to 4.4 wt.% (Figure 3(B1,B2)). The composite E-ZFA-Fe_3_O_4_ showed the increase in iron content (~25.9 wt.%) compared with bare zeolite (Figure 3(C1,C2)), proving the effective modification of the sample with magnetic nanoparticles. In the case of E-ZFA-Ag-Fe_3_O_4_, both iron and silver elements could be observed (23.9 and 2.8 wt.% respectively) (Figure 3(D1,D2)). The TEM analysis also confirmed the formation of Ag^0^ NPs and Fe_3_O_4_ NPs within the microstructure of composites, as shown in Figure 4.

**Figure 5:**
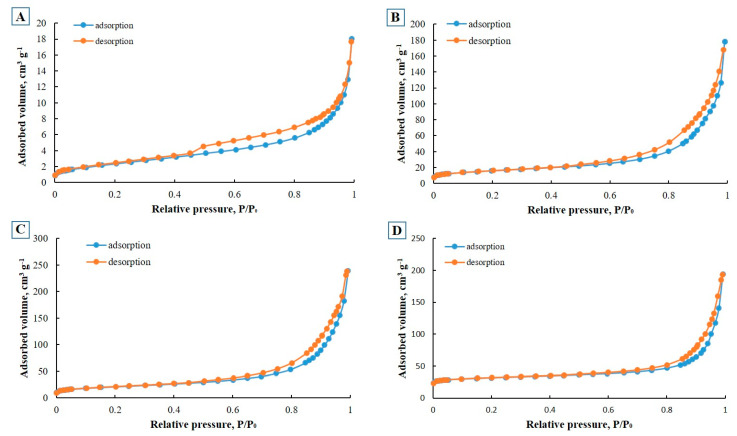
Nitrogen adsorption isotherms of the E-CFA (**A**), E-ZFA-500 (**B**), E-ZFA-600 (**C**), and E-ZFA-700 (**D**).

The surface area of zeolites also plays an important role in determining adsorption capacity. The cumulative surface area refers to the surface area contributed by all the pores. The typical surface area of synthetic zeolites obtained from CFA is in the range of 40–260 m^2^/g [32,33,34]. The surface area can be divided into two groups: the inner surface area and the outer surface area. The latter is very small compared to the internal surface area, so the internal surface area determines the degree of adsorption of the sample. The outer surface area indicates the degree of contact between the adsorbate and the zeolite and determines the rate of transfer of adsorbate molecules from aqueous solution to the zeolite structure. The low-temperature nitrogen adsorption data for the E-CFA and E-ZFAs were analyzed by BET methods, as presented in Figure 5.

The results obtained by the method of low-temperature nitrogen adsorption showed that the surface areas of the initial E-CFA show comparable values with the literature [35], and the calculated volumes of macropores and mesopores demonstrate a large proportion of the total pore volume related to macropores. The results of low-temperature nitrogen porosimetry show that all the synthesized zeolites have a high specific surface area and pore volume compared to the initial CFA, which was to be expected. This indicates that the process of synthesis of zeolites with a crystal structure was successful, and contributed to the formation of mesopores and micropores, which thus led to a sharp increase in surface area and pore volume.

The results of the BET surface analysis show that the CFA-derived zeolites and nanocomposites have a 5- to 14-fold increase in specific surface area. Pristine E-CFA showed the lowest surface area of 8.7 m^2^/g, while the largest surface area of 128.7 m^2^/g was recorded for E-ZFA-Ag. The substantial increase in the E-ZFA-Ag is due to the formation of fine Ag NPs that expanded the total surface. Modification of synthetic zeolites with Fe_3_O_4_ NPs noticeably reduced the surface area, which might be because of the partial blockage of micro- and mesopores. Synthetic zeolite modified with a combination of E-ZFA-Ag-Fe_3_O_4_ nanoparticles displayed an average surface area of 86.9 m^2^/g. The pore sizes of zeolite and nanocomposites, as shown in Table 4, range from 0.6 to 4.8 nm, and are also correlated with the surface area and pore volume, as well as with the removal performance.

**Figure 6:**
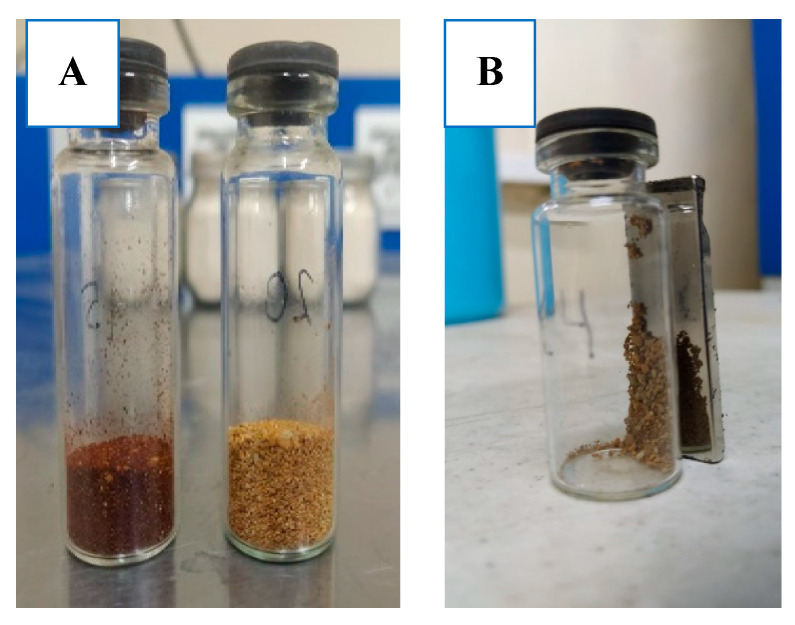
Representative images of freshly synthesized E-ZFA-Ag (right) and E-ZFA-Fe_3_O_4_ (left) nanocomposite (**A**) and magnetic properties of the E-ZFA-Fe_3_O_4_ (**B**).

All studied samples of zeolites are characterized by the presence of micropores and mesopores, as well as an increased specific surface area. The lowest surface area was characteristic of the parent E-CFA, while all synthetic zeolites obtained from E-CFA showed a relatively high surface area and pore volume. The absence of macropores in the studied samples indicates the possibility of using these zeolites for the adsorption of heavy metals from water. Representative images of the synthesized CFA-derived E-ZFA-Ag and E-ZFA-Fe_3_O_4_ nanocomposites, as well as the qualitative examination of the magnetic properties of the E-ZFA-Fe_3_O_4_, are shown in Figure 6.

### 2.2. Adsorption Kinetics

**Figure 7:**
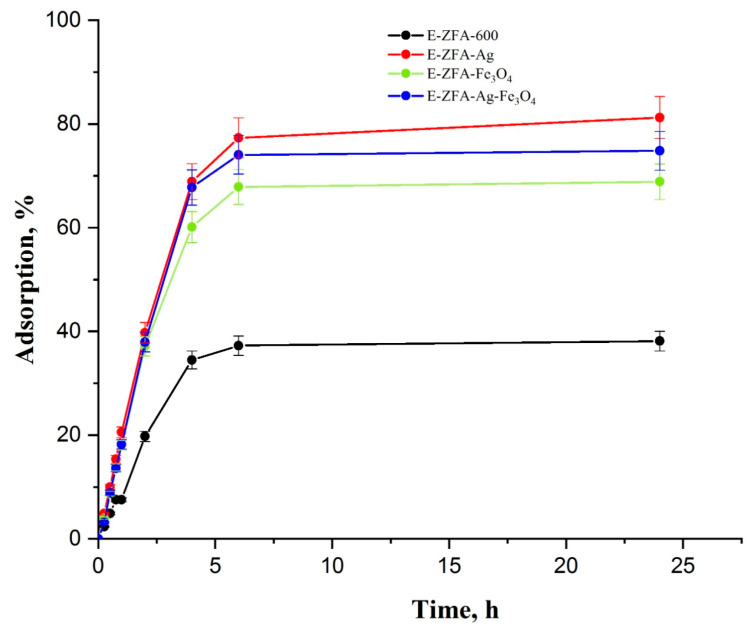
The adsorption kinetics of parent zeolite E-ZFA-600 and modified nanocomposites.

The preliminary adsorption kinetics results show a significantly higher removal of mercury from the solution compared to the parent zeolite, as presented in Figure 7. The minimum values were recorded for E-ZFA-600, which revealed the lowest adsorption removal of only 38.1% after 24 h. Nanocomposites demonstrated substantially improved removal efficiencies as opposed to pristine zeolite. For instance, Fe_3_O_4_ NP-impregnated E-ZFA-Fe_3_O_4_ removed 18.1% of mercury ions during the first hour of adsorption kinetics, and reached significantly improved removal efficiency of 68.9% after the whole 24 h period. The latter is most presumably related to a slight chemical interaction of mercury ions with impregnated Fe_3_O_4_ NPs, as observed previously [36].

In contrast to these samples, the samples modified with Ag^0^ NPs and a combination of Ag-Fe_3_O_4_ NPs, i.e., E-ZFA-Ag and E-ZFA-Ag-Fe_3_O_4_, showed noticeably improved removal efficiencies. The residual concentrations of mercury ions in water for the first hour of adsorption by these samples showed similar values, wherein the removal efficiencies corresponded to 20.5% and 18.2%, respectively. However, after 24 h of adsorption kinetics, the Ag^0^ NP-impregnated E-ZFA-Ag removed 81.2% of the mercury ions, which is 2-fold higher than the removal of the parent zeolite E-ZFA-600. The silver-magnetite nanocomposite E-ZFA-Ag-Fe_3_O_4_ showed 74.8% removal efficiency, which is 36.7% and 5.9% higher than that for E-ZFA-600 and E-ZFA-Fe_3_O_4_, but 6.4% lower than that for E-ZFA-Ag nanocomposite. Time-dependent adsorption capacities of 24 h points for all studied samples ranged from 7.8 mg/g to 16.4 mg/g by following this trend: E-ZFA-Ag > E-ZFA-Ag/Fe_3_O_4_ > E-ZFA-Fe_3_O_4_ >> E-ZFA-600.

The predominant mechanism involved in pristine zeolite and Fe_3_O_4_ NP-impregnated nanocomposite is most probably physisorption with a substantial involvement of chemisorption in the latter due to the ferric oxyhydroxide FeO(OH)---Hg interaction, as was previously observed [36]. On the other hand, the Ag^0^ NP-modified nanocomposite clearly revealed the prevalence of the chemisorption between the mercury ions and Ag^0^ NPs (Hg---Ag). This is further enhanced with physisorption facilitated by the porous structure of zeolite, allowing Hg^2+^ ions to diffuse faster and increasing removal capacity. According to the results, it could be tentatively hypothesized that physical adsorption is predominant in E-ZFA-600, while the E-ZFA-Fe_3_O_4_, E-ZFA-Ag-Fe_3_O_4_, and E-ZFA-Ag nanocomposites undergo a combination of physical adsorption owing to a porous structure and chemical adsorptions due to the impregnated Ag^0^ and Fe_3_O_4_ NPs, with the latter adsorption mechanism being predominant. The fast and efficient removal of the nanocomposites, as well as the adsorption isotherm profiles, confirm the proposed mechanisms, which in turn corroborate with porosimetric analysis. The previous speciation studies of mercury ions under different experimental conditions and adsorption kinetics and equilibrium studies also discovered similar mechanisms with Ag^0^ and Fe_3_O_4_ NP-impregnated nanocomposites, as found elsewhere [22,36].

These results firmly confirm the feasibility of internal modifications of zeolite with Ag^0^ and Fe_3_O_4_ NPs to enhance both the removal efficiency and separation performance from water after the usage of nanocomposites.

### 2.3. Adsorption Isotherms

**Figure 8:**
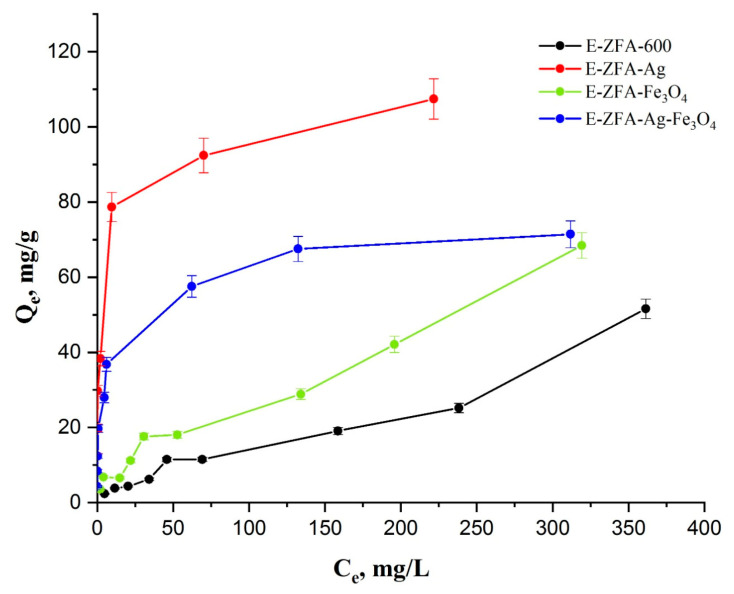
The adsorption isotherms of parent zeolite E-ZFA-600 and modified nanocomposites.

In order to elaborate on the adsorption performance of the bare and modified zeolites, the batch adsorption isotherm was studied (Figure 8). According to the results, the pristine zeolite showed the lowest equilibrium adsorption capacity of 51.5 mg/g for Hg^2+^ removal from water, which is prevalently due to physical adsorption. In the case of modified nanocomposites, the impregnation of Fe_3_O_4_ NPs improved the removal capacity of the pristine zeolite by around 32.1% and reached an adsorption capacity value of 68.4 mg/g. This improvement could be explained by the formation of ferric oxyhydroxide FeO(OH) under acidic conditions, which allowed the chemical adsorption of Hg^2+^.

The highest adsorption capacity was achieved by E-ZFA-Ag, of 107.4 mg/g, which might be due to the amalgam formation reaction between Hg---Ag. Similar results were identified by Katok and co-authors [37,38] while studying the hyperstoichiometric interaction between silver and mercury at the nanoscale. Nanocomposites containing both Ag^0^ and Fe_3_O_4_ NPs demonstrated a lower adsorption capacity (71.4 mg/g) compared to E-ZFA-Ag, but a slightly higher adsorption capacity than E-ZFA-Fe_3_O_4_, probably due to a partial blockage of Ag^0^ NPs, which restricted the impregnation of Fe_3_O_4_. However, this clearly demonstrated that impregnation of Ag^0^ NPs significantly improved the adsorption capacity of the pristine zeolite, while the inclusion of Fe_3_O_4_ NPs provided a magnetic property for an efficient post-adsorption separation. In addition to this, our previous work on desorption studies of Hg^2+^ from Ag^0^ NP-containing nanocomposites [24] showed a prolonged stability over 12 days under neutral and strong acidic pH conditions of less than 0.9%. These results further demonstrate the advantages of nanocomposites over a pristine CFA-derived synthetic zeolite in terms of stability. Table 5 summarizes the adsorption capacities of CFA-derived zeolites and nanocomposites synthesized in this work, as well as literature data.

Both the Langmuir and Freundlich models were used to determine the adsorption performance of materials (Table 6). It is interesting to note that different processes are observed for composites. For samples of the pristine E-ZFA-600 and Fe_3_O_4_ NP-modified zeolite E-ZFA-Fe_3_O_4_, the correlation coefficient of the Freundlich model is higher than that for the Langmuir model (0.9631 and 0.9424 against 0.8639 and 0.8399, respectively), which indicates a multilayer adsorption mechanism. In the case of zeolites modified with Ag^0^ NPs (E-ZFA-Ag) and the combination of Ag^0^ and Fe_3_O_4_ NPs (E-ZFA-Ag-Fe_3_O_4_), the R^2^ values for the Langmuir model are close to 1, and the model values of the maximum sorption capacity are close to the experimental values. However, based on the average values of both studied models for all samples, the Freundlich model is considered more reliable.

## 3. Materials and Methods

### 3.1. Materials 

CFA samples were collected from electrostatic precipitators of the coal-fired power plants in Nur-Sultan and Oskemen cities at maximum electrical load. CFA samples taken in Nur-Sultan were labeled as E-CFA, since they use coal from the Ekibastuz deposit. All CFA samples were used in their original form, without preliminary washing and sieving. Prior to the experiment, CFA samples were homogenized and dried in an oven at 120 °C for 6 h. After that, CFA samples were placed into sealed containers for use in the synthesis of zeolites and their characterization. Silver nitrate (AgNO_3_, 98%, St. Louis, MO, USA), iron (III) chloride (FeCl_3_, anhydrous, 99%, Fisher Scientific, Loughborough, UK), and iron (II) sulfate heptahydrate (FeSO_4_, ACS grade, 99%, St. Louis, MO, USA) were used to impregnate Ag^0^ and Fe_3_O_4_ NPs into the porous structure of CFA-derived zeolites. Sodium borohydride (NaBH_4_, 98%, Fisher Scientific, Loughborough, UK) was used as a reducing agent for reduction of silver ions, while ammonia solution (NH_4_OH, 25%, Fisher Scientific, Loughborough, UK) was used as a strong reduction medium during the synthesis of Fe_3_O_4_ NPs.

### 3.2. Synthesis of Zeolite

Synthesis of zeolites was carried out using sodium hydroxide (NaOH, chemically pure) in powder form. A certain amount of NaOH in the ratio of CFA (1.2:1) was weighed, thoroughly mixed, and placed in a refractory crucible to fuse under 500 °C, 600 °C, and 700 °C for 1 h to obtain a melted glass phase containing aluminosilicate species. The obtained samples were further crystallized under 120 °C for 6 h to produce crystalline zeolitic structures. The final CFA-derived zeolites were thoroughly washed with deionized water until no pH changes were observed, dried at 90 °C for 12 h, and stored in a sealed container for further modifications and analysis. Zeolites synthesized from the E-CFA were named E-ZFA-500, E-ZFA-600, and E-ZFA-700 depending on the fusion temperature used during synthesis. 

### 3.3. Synthesis of Nanocomposites

The modification of the obtained zeolites was carried out using iron ions (Fe^2+^ and Fe^3+^) to impart magnetic properties for easy separation purposes and to impregnate silver ions in order to impregnate Ag^0^ NPs to improve the affinity towards target pollutant Hg^2+^ ions. 

#### 3.3.1. Magnetite NP-Impregnated Zeolitic Nanocomposites

Nanoparticles were impregnated by a co-precipitation method followed by a hydrothermal treatment. For this purpose, 1.77 g of FeCl_3_ × 4H_2_O and 2.48 g of FeSO_4_ × 7H_2_O were added to the reaction container and mixed in 10 mL of deionized water under 700 rpm to fully dissolve the salts. After dissolving the iron salts, 5 g of zeolite was added and 10 mL of ammonia was slowly added to initiate the reduction and formation of Fe_3_O_4_ NPs within the zeolitic structure. After 2 min, the reaction mixture was transformed into a Teflon-lined autoclave reactor under 120 °C for 12 h. The reaction mixture was cooled down to room temperature before filtration and purification with distilled water until reaching a neutral pH of the effluent. Finally, the obtained magnetite modified zeolite was dried at 120 °C for 6 h in a drying oven, cooled to room temperature, and stored in sealed vials for further use. 

#### 3.3.2. Silver NP-Impregnated Zeolitic Nanocomposites

Silver NPs were impregnated into zeolitic structures via ion exchange followed by the reduction method. For this purpose, 0.158 g of AgNO_3_ was dissolved in 20 mL of deionized water in the reaction container. After that, 2 g of zeolite was added to the solution in a reaction container and rigorously mixed at 700 rpm. In order to initiate the reduction of silver ions, 0.2 g of NaBH_4_ was slowly added to this mixture for 5 min. The reaction contained was wrapped with aluminum foil to avoid formation of silver oxide and left overnight for 12 h. After that, the obtained crude product was washed with deionized water and dried in an oven at 120 °C for 6 h. 

#### 3.3.3. Silver-Magnetite NP-Impregnated Zeolitic Nanocomposites

Silver-magnetite NP-impregnated zeolitic nanocomposites were synthesized using the two abovementioned methodologies by a sequence of magnetite NP impregnation followed by silver NP impregnation. The sequence was based on the activity of the metals, which allowed a possible metal replacement reaction to be avoided. 

### 3.4. Characterization of Materials

The powder forms of the raw CFA samples, as well as those of the produced zeolites and nanocomposites, were analyzed for chemical composition using a standardless X-ray fluorescence (XRF, PANalytical, Malvern, UK) spectrometer. The phase composition of samples was examined using X-ray diffraction (XRD, Rigaku, Tokyo, Japan) using the Rigaku SmartLab diffraction system with CuKβ radiation at 40 kV and 30 mA. The morphological characteristics of obtained zeolites and nanocomposites were studied using a scanning electron microscope (JEOL 6380LV, JEOL, Tokyo, Japan) at 20 kV, equipped with a backscattered electron detector. The elemental composition via a spot and area analysis was conducted using a Si(Li) Energy-Dispersive X-ray spectrometer (INCA X-sight, Oxford Instruments, Oxford, UK) that was connected to the SEM. The nanoscale investigation was performed with a high-resolution JEOL JEM-2100 LaB6 transmission electron microscope (HR-TEM, JEOL, Tokyo, Japan), operating at 200 kV. The pore size distribution and specific surface area analysis were performed by nitrogen adsorption at −196 °C using Autosorb-1 (Quantachrome, Hook, UK). The pore size distribution was calculated using the BJH method, while the specific surface area was determined using the BET method. 

### 3.5. Batch Adsorption Kinetics 

Adsorption kinetics were studied using 40 mL of a freshly prepared aqueous solution of Hg^2+^ with an initial concentration of 50 mg/L and an adsorbent dosage of 0.1 g. Concentrated nitric acid was used to adjust the pH of a solution to 2.5, which allowed the precipitation and hindering effects of mercury complexes to be avoided. The details of speciation studies using Medusa software and the possible formation of mercury complexes under certain conditions can be found elsewhere [22]. The experiments were carried out at an ambient temperature and static conditions. Each kinetics point (1 h, 2 h, 4 h, 8 h) was analyzed using 20–25 µL of aliquot that was taken from adsorption containers. The removal efficiency of the adsorbent at each adsorption kinetics point was calculated using the concentration differences between the initial and adsorption kinetics point. Hg^2+^ solution without addition of adsorbents, but adjusted with concentrated nitric acid, was used as a control. The measurements were conducted in duplicate with an average standard deviation below 2.5%. 

### 3.6. Batch Adsorption Isotherm

A quantity of 25 mg of bare zeolite or composites was mixed with 10 mL of Hg^2+^ solution at different initial concentrations from 10 to 500 mg/L in 15 mL plastic tubes. The solutions with adsorbents were shaken at 100 rpm on an orbital shaker (Rotamax 120, Heidolph) at room temperature for 24 h. The quantity of the adsorbed metal ions was calculated using Equation (1):

(1) $$q_{max}=\frac{\left(C_{i}-C_{eq}\right)V}{m}$$

where *q_max_* (mg/g) is the maximum adsorption capacity (mg/g), *C_i_* and *C_eq_* are the initial and equilibrium metal concentrations (mg/L), respectively, *V* represents the total solution volume (mL), and *m* represents the total mass (g) of adsorbent used. All experiments were performed in duplicate.

Langmuir and Freundlich isotherm models were used to evaluate the fitness of the experimental data to study the adsorption behavior on both homogeneous and heterogeneous surfaces. The linearized form of the Langmuir isotherm is expressed in Equation (2) [41]:

(2) $$\frac{C_{eq}}{q_{eq}}=\frac{1}{q_{max}K_{L}}+\frac{C_{eq}}{q_{max}},$$

where *C_eq_* (mg/L) is the equilibrium concentration of Hg^2+^ in the water phase, *q_eq_* and *q_max_* (mg/g) are the equilibrium and maximum removal capacity, and *K_L_* (L/mg) is the Langmuir constant. The linear form of the Freundlich isotherm is expressed in Equation (3) [42]:

(3) $$logq_{eq}=logK_{F\;}+\frac{1}{n}logC_{eq}$$

where *C_eq_* (mg/L) and *q_eq_* (mg/g) are the equilibrium concentrations’ adsorption capacity, while *n* (dimensionless) and *K_F_* are Freundlich constants. 

## 4. Conclusions

In conclusion, this study successfully demonstrated the synthesis of zeolites derived from CFA using the fusion-assisted alkaline hydrothermal method. The obtained zeolites exhibited the formation of analcime and sodalite, confirming the successful synthesis of zeolitic frameworks. Additionally, the incorporation of Fe_3_O_4_ and Ag^0^ NPs further enhanced the properties of the zeolites. The characterization analyses, including XRD and XRF composition analyses, provided conclusive evidence of the presence of Fe_3_O_4_ and Ag^0^ NPs in the synthesized nanocomposites. The microporous structure of the zeolites and nanocomposites was confirmed through nitrogen porosimetric analysis, with BET surface areas ranging from 48.6 to 128.7 m^2^/g and pore sizes ranging from 0.6 to 4.8 nm. The changes observed in the porosimetric characteristics of the zeolites after modification can be attributed to the successful impregnation of Fe_3_O_4_ and Ag^0^ NPs. This indicates that the modification with nanoparticles significantly alters the overall porous structure of the zeolites, which corroborates with the removal performance. Furthermore, the synthesized zeolite composites exhibited promising adsorption capacities for mercury ions in aqueous solutions. Specifically, the composites containing Ag^0^ NPs demonstrated the highest adsorption capacity, reaching 107.4 mg of Hg^2+^ per gram of composite. The nanocomposites modified with Fe_3_O_4_ and Ag/Fe_3_O_4_ NPs also exhibited considerable adsorption capacities, with values of 68.4 mg/g and 71.4 mg/g, respectively. These findings highlight the potential of the fusion-assisted method for producing synthetic zeolites and nanocomposites from CFA, which can be utilized for various environmental applications. The ability of the synthesized composites to effectively remove Hg^2+^ from water solutions suggests their potential for mitigating water pollution and addressing environmental concerns. The study contributes to the expanding field of sustainable materials synthesis and environmental remediation by providing valuable insights into the synthesis of zeolites from CFA and their enhanced properties through nanoparticle modifications. Further research can explore additional applications and optimize the synthesis process for improved efficiency and environmental impact.

## Figures and Tables

**Table 1:** Fusion-assisted synthesis of CFA-derived zeolites for the removal of heavy metals from water.

Synthetic Zeolite Type	* T_f_ °C	* t_f_ [h]	* T_c_ [°C]	* t_c_ [h]	Additional Synthesis Step	NaOH:CFA Ratio	Heavy Metals	Conc. [mg/L]	Removal [%]	Ref.
Zeolite X	550	1.5	90	15	Hydrothermal treatment	2:1	Ni^2+^	20	90	[15]
Sodalite	550	1	105	24	Hydrothermal treatment	2:1	Pb^2+^ Cu^2+^ Mn^2+^	1.8 7.5 5.7	93 95 78	[16]
Zeolite	450	4	90	12	Microwave digestion	5:1	Cd^2+^	20	98	[17]
Zeolite Na-P1	550	2	100	12	Hydrothermal treatment	1:1	Cr^3+^	100	-	[18]
Zeolite A	650	2	100	24	Hydrothermal treatment	1:1	Ni^2+^	200	94	[19]
Zeolite Na-X	550	-	90 105	4 1	Hydrothermal treatment	2:1	Cd^2+^ Pb^2+^	50 100	-	[20]
Al_2_O_3_-NaA zeolite	600	2	RT	12	Hydrothermal treatment	1:0.7	Pb^2+^	50	99	[21]

* T_f_—fusion temperature; t_f_—fusion time; T_c_—crystallization temperature; t_c_—crystallization time.

**Table 2:** Chemical composition of E-CFAs and E-ZFAs produced at different fusion temperatures (wt.%).

Elements	E-CFA	E-ZFA-500	E-ZFA-600	E-ZFA-700
O	-	9.369 ± 0.128	9.978 ± 0.163	6.326 ± 0.162
Na	-	2.455 ± 0.076	2.138 ± 0.058	1.505 ± 0.045
Mg	0.140 ± 0.009	0.232 ± 0.002	0.167 ± 0.023	0.162 ± 0.028
Al	14.165 ± 0.046	14.632 ± 0.245	14.024 ± 0.245	12.934 ± 0.845
Si	46.954 ± 0.519	31.150 ± 0.845	31.471 ± 0.724	34.190 ± 0.923
P	0.941 ± 0.012	1.536 ± 0.063	1.637 ± 0.102	1.874 ± 0.148
S	0.274 ± 0.004	0.361 ± 0.086	0.359 ± 0.054	0.047 ± 0.004
Cl	0.747 ± 0.024	0.713± 0.094	0.626 ± 0.073	0.107 ± 0.012
K	1.567 ± 0.126	0.377 ± 0.068	0.293 ± 0.028	1.081 ± 0.145
Ca	6.499 ± 0.175	8.398 ± 0.284	8.216 ± 0.438	8.147 ± 0.628
Fe	22.275 ± 0.481	24.370 ± 0.172	24.537 ± 0.984	26.627 ± 0.823

**Table 3:** XRF elemental composition of Ag^0^ and Fe_3_O_4_ NP-modified zeolites (wt.%).

Elements	E-ZFA-Ag	E-ZFA-Fe_3_O_4_	E-ZFA-Ag-Fe_3_O_4_
Na	1.982 ± 0.109	1.341 ± 0.359	1.796 ± 0.157
Al	5.476 ± 0.358	6.355 ± 0.823	6.285 ± 0.643
Si	12.623 ± 0.636	12.424 ± 1.344	13.203 ± 0.823
K	0.302 ± 0.018	0.113 ± 0.012	0.201 ± 0.009
Ca	2.629 ± 0.110	2.308 ± 0.228	2.718 ± 0.196
Ti	0.771 ± 0.017	0.678 ± 0.018	0.794 ± 0.037
Mn	0.954 ± 0.048	0.113 ± 0.002	0.107 ± 0.004
Fe	6.002 ± 0.227	11.552 ± 1.018	8.760 ± 0.182
Ag	2.318 ± 0.365	-	1.259 ± 0.596
Other *			

* Ni, Sr, Zr, Ba, Eu, Pb, Mg, S are less than 0.0005%.

**Table 4:** The porosimetric analysis of the E-CFAs and E-ZFAs.

Sample Name	BET Surface Area (m^2^/g)	Pore Size (nm)	Total Pore Volume (cm^3^/g)	Micropore Volume (cm^3^/g)
E-CFA	8.7	1.9	0.027	0.003
E-ZFA-600	74.9	4.8	0.076	0.023
E-ZFA-Fe_3_O_4_	48.6	1.0	0.015	0.013
E-ZFA-Ag	128.7	0.6	0.092	0.061
E-ZFA-Ag-Fe_3_O_4_	86.9	0.7	0.057	0.036

**Table 5:** Equilibrium studies on Hg^2+^ removal from water.

Adsorbent Type	Adsorption Capacity [mg/g]	pH Range	Initial Concentration [mg/L]	Ref.
E-ZFA-600	51.5	2.5	490	This work
E-ZFA-Ag	107.4	2.5	490	This work
E-ZFA-Fe_3_O_4_	68.4	2.5	490	This work
E-ZFA-Ag-Fe_3_O_4_	71.4	2.5	490	This work
CFA-derived zeolite	0.3	2.5	10	[35]
Fe_3_O_4_-Ag^0^ nanocomposite	71.3	6.5	200	[36]
CFA-derived zeolite	5.1	5-6	575	[39]
Ag-doped CFA-derived zeolite	5.0	5-6	575	[39]
Fe_2_O_3_@SiO_2_ thin films	126.0	7.0	336	[40]

**Table 6:** Isotherm models’ data for adsorption of Hg^2+^ by zeolites.

	Experimental	Langmuir Model			Freundlich Model			
	q_max_, mg/g	q_max_, mg/g	K_L_	R^2^	n	K_F_	R^2^	
E-ZFA-600	51.5	35.2	0.0086	0.8639	1.4684	1.4521	0.9631	
E-ZFA-Fe_3_O_4_	68.4	48.3	0.0168	0.8399	1.8646	2.4005	0.9424	
E-ZFA-Ag	107.4	107.5	0.3298	0.9976	4.6970	39.884	0.9471	
E-ZFA-Ag-Fe_3_O_4_	71.4	71.9	0.1601	0.9981	4.1425	20.247	0.9677	

## Data Availability

The data presented in this study are available on request from the corresponding author.
